# Clinical Manifestations of COVID-19 in the Feet: A Review of Reviews

**DOI:** 10.3390/jcm10102201

**Published:** 2021-05-19

**Authors:** Ana Maria Jimenez-Cebrian, Aurora Castro-Mendez, Blanca García-Podadera, Rita Romero-Galisteo, Miguel Medina-Alcántara, Irene Garcia-Paya, Joaquín Páez-Moguer, Antonio Córdoba-Fernández

**Affiliations:** 1 Department of Nursing and Podiatry, Faculty of Health Sciences, University of Málaga, 29071 Málaga, Spain; blancagarciapodadera1997@gmail.com (B.G.-P.); migmedalc@uma.es (M.M.-A.); irenegpaya@uma.es (I.G.-P.); joaquinpaez@uma.es (J.P.-M.); 2 Biomedical Research Institute (IBIMA), 29010 Málaga, Spain; 3 Department of Podiatry, Faculty of Nursing, Physiotherapy and Podiatry, University of Sevilla, 41009 Sevilla, Spain; acordoba@us.es; 4 Department of Physiotherapy, Faculty of Health Sciences, University of Málaga, 29071 Málaga, Spain; rpromero@uma.es

**Keywords:** COVID-19, foot, clinical manifestations, signs and symptoms, review of reviews

## Abstract

There is a lack of scientific evidence about the severe acute respiratory syndrome coronavirus 2 (SARS-CoV-2). The clinical manifestations are not thoroughly understood; classically, the virus manifests itself at the pulmonary level but can manifest at other levels. To the best of our knowledge, systematic reviews and non-systematic reviews about COVID-19 symptoms in the feet have not been published. The aim of this review of reviews was to analyze and synthesize the published reviews on manifestations of COVID-19 at the foot level. Methods: a review of reviews was conducted; the eligibility criteria included studies published in English or Spanish, involving children and adults with COVID-19, and reporting foot manifestations. PubMed, SciELO, Science Direct, Cochrane Database of Systematic Review, and Google Scholar were analyzed. Two authors independently performed the screening and quality assessment of the studies with AMSTAR 1, and finally, nine reviews were analyzed (one systematic and eight narratives studies). The main clinical manifestations at the foot level in patients with COVID-19 were vascular (edema, exanthems, chilblains, ischemia, and distal necrosis), dermatological (vesicular, maculopapular, papulosquamous, urticarial skin breakouts, and recurrent herpes), and neurological (muscular weakness in lower limbs, paresis, areflexias, ataxia, and difficulty walking). Erythema pernio or “COVID toes” was shown as the most characteristic lesion of this disease, especially in asymptomatic children and young people, so this typical manifestation may be considered important in patients who are positive for COVID-19. This finding does not allow for strong conclusions due to the scarce literature and methodological quality in this regard. Future studies are necessary.

## 1. Introduction

In December of 2019 a new virus of zoonotic origin, called coronavirus (SARS-CoV-2), was reported in Wuhan, China; it expanded and quickly resulted in a global pandemic called Coronavirus Disease 2019 (COVID-19), declared by the WHO on 12 March 2020 [1,2].

The COVID-19 infection often affects the lungs and can manifest into an infectious pneumonia [1]. Although nucleic acid detection is the determinant for identifying COVID-19 infection, and more rapid detection kits for the novel coronavirus have come into mass production, computed tomography and computer aided diagnosis help [3,4,5]. In turn, new manifestations of the virus affecting the gastrointestinal, cardiovascular, dermatological, and nervous system appeared [4,5], generating disorders for those infected [4]. Heart disease is a disorder present in patients with COVID-19; monitoring of patients with cardiac arrhythmias is important in this infection [6].

Patients with COVID-19 (COVID-19+) may develop symptoms such as fever, cough, pharyngeal pain, abdominal pain, diarrhea, conjunctivitis, muscle fatigue, and pneumonia [1,6] and may be left with serious side effects or even die [7]. Symptomatology is diverse and depends on whether the patient is a child or an adult [6]; fever, cough, rhinorrhea, vomiting, diarrhea, and myalgias are more common in infants, while their respiratory systems are less severe [8]; children account for 1–5% of asymptomatic cases [2].

Based on severity, this disease can be divided into five clinical subtypes: asymptomatic infection, mild disease, characteristic clinical case, severe clinical case, and critically severe case [6]. There are numerous asymptomatic cases to date and we do not yet understand why, so it is considered relevant to identify the clinical manifestations of COVID-19, specifically skin manifestations, because they may be a typical indicator of COVID-19 infection in asymptomatic subjects [9]. Recently, skin lesions have been described as possible manifestations of COVID-19. There is great attention to the clinical implications of acute acral lesions (similar to pernio erythema or chilblains) in asymptomatic or slightly symptomatic patients. These dermatological lesions can be considered indicators of indolent evolution and good prognosis in those infected with SARS-CoV-2 [10].

It has been argued that in some patients positive for COVID-19 (COVID-19+) there are widespread manifestations at the skin level or in isolation in lower limbs and/or feet [2,11,12,13,14,15]. Different authors agree that in children and young adults with suspected COVID-19 infection, reddish purple nodules have been observed on the toes, similar in appearance to pernio erythema or chilblains (“COVID toes”) [15,16,17]. They also emphasize that possible dermatological complications are more common and serious in diabetic patients [18]. The distribution of these skin lesions in patients with COVID-19 is mainly located on the torso (66.7%) and the hands and feet (19.4%). In 12.5% of these subjects, some symptoms in the feet correspond to skin lesions that appeared prior to respiratory symptoms or diagnosis of COVID-19. In 18.1% of patients, the lesion onset time was unknown, 74% of patients developed the skin pathology in the 7 days prior to their diagnosis of COVID-19, and 6% in the 7 days after their diagnosis. Healing of skin lesions usually occurred approximately 10 days after their appearance [9].

Involvement of the vascular and neurological systems are often found; the immune response triggered against SARS-CoV-2 infection can result in severe vascular alterations due to endothelial cell dysfunction and activation of clotting pathways. Some cases of gangrene have been reported in patients with COVID-19, possibly due to thrombotic events, with distal extremities being the most susceptible and most commonly affected in hospitalized patients with underlying diabetes or peripheral vascular disease. This acral ischemia in severely ill patients with COVID-19 should not be confused with chilblain-like lesions in young people [19,20,21]. At the neurological and systemic level, these manifestations may be due to the massive immune response produced. The susceptibility of patients with COVID-19 to nerve damage may be secondary to the state of hyperinflammation induced by the virus [22]. The actual incidence of vascular and neurological complications is still uncertain [20].

There are reviews of the literature in this field of study, but they are not specific to the feet, which justifies our proposed review, allowing us to compare and contrast the published reviews and provide a synthesis to the following question: What clinical manifestations can appear in the feet of patients with COVID-19?

Therefore, this review of reviews aimed to analyze and synthesize the current state of knowledge about specific and localized clinical manifestations in the feet of patients with COVID-19 using review of reviews research.

Since early detection of SARS-CoV-2 infection is critical to preventing its spread, and since recent studies show that at the foot level there are clinical manifestations characteristic of COVID-19 infection, it is of great importance to analyze the scientific literature about the typical manifestations of this disease in this anatomical region and highlight if there is a typical marker that will allow for the identification of COVID-19 infection.

## 2. Methods

A detailed protocol was registered for the review in the International Prospective Register of Systematic Reviews (PROSPERO CRD42021234395). The Preferred Reporting Items for Systematic Reviews and Meta-Analyses (PRISMA) was followed to conduct this review of reviews.

### 2.1. Eligibility Criteria

The eligibility criteria were based on the terms Population, Exposure, and Outcome (PEO) (Table 1).

#### 2.1.1. Inclusion Criteria

##### Case Study Design: Participants

A methodological design for review was chosen to summarize and analyze the evidence available on this subject. A review of reviews was carried out, systematically and non-systematically, including observational studies. To date, research had been conducted on case studies of low methodological quality and reviews focused on published and mostly narrative case studies.

Reviews considered were the ones in which participants were symptomatic or asymptomatic subjects confirmed with COVID-19 or with clinical suspicion of COVID-19.

##### Outcome Measures

The reviews included in our review were those presenting results of clinical manifestations (signs and symptoms) in the feet of patients with COVID-19. All signs and symptoms in feet were eligible for this review

##### Language

Reviews published in English or Spanish were eligible.

#### 2.1.2. Exclusion Criteria

Reviews that did not meet the predefined criteria mentioned above and reviews that were not carried out on humans.

### 2.2. Research Methods for Identifying Other Studies

An initial search was carried out in the PROSPERO (International Prospective Register of Systematic Reviews) database with the intention of checking whether this topic had previously been addressed by registered reviews. No previous reviews were found on the subject of this article.

Systematic research identified relevant systematic and non-systematic reviews based on the criteria for inclusion in the following electronic databases: PubMed, SciELO, Science Direct, Cochrane Database of Systematic Reviews, and the Google Scholar gray literature database. The keywords searches were assigned to Medical Subject Headings (MeSH). The complete search strings can be found in Table 2. All included studies were published between April 2020 and February 2021.

### 2.3. Data Collection, Analysis, and Extraction

The search strategy was carried out by filtering by year, language, and people and selections were made after reading the title, the summary, and finally after reading the full text. Duplicate articles were deleted using a bibliographic management software (Mendeley desktop v1.17.4) and hand verified. Eligible studies were selected using a multi-step approach (title reading, summary, and full-text evaluation). To this effect, two blinded researchers (B.G.-P. and A.M.J.-C.) analyzed titles and summaries independently. Each researcher then evaluated the full texts according to the inclusion criteria. Disagreements between these two reviewers were resolved during the consensus session with a third author (A.C.-M.). If necessary, sending an email to the original authors to learn more about the findings of the study was also planned, but this option was not necessary for any study.

### 2.4. Data Management

In the second screening stage, the two independent reviewers (B.G.-P. and A.M.J.-C.) performed full-text reading for the extraction of the following information from each article:

1. First author and year of publication;
2. Title;
3. Type of review (systematic or narrative);
4. Study designs (from studies included in the review, all of which were descriptive);
5. Number of included studies (studies included in the review);
6. Results: clinical manifestations in the feet of patients with COVID-19.

### 2.5. Quality Assessment Tools

Lastly, two independent reviewers (B.G.-P. and A.M.J.-C.) evaluated the methodological quality of the included reviews, for which the updated version of AMSTAR 1 was used, a tool used to estimate the methodological quality of the reviews. It consists of 11 items with a good validity of construct, content, and reliability [23,24].

The overall final value of each review was considered to be high, moderate, low, or critically low. In case of disagreements, a consensus session was held with the third reviewer (A.C.-M.).

## 3. Results

### 3.1. Review Selection

The electronic search initially resulted in *n* = 2126 citations. A total of 1762 duplicate studies were excluded; after removing duplicates (*n* = 326) and after the review of the title and summary, 295 studies were removed (*n* = 69).

Subsequently, 59 studies were excluded after a full-text review as they did not meet the inclusion criteria (37 for not responding to the study objectives and 22 for being exclusively case study reviews). Finally, 10 reviews were selected and read for quality assessment. After the AMSTAR 1 criteria were applied by two researchers independently, 1 article [25] was excluded for only meeting 7 of the 11 criteria to be evaluated (7/11), which is a low-quality indicator. A total of nine articles [6,7,8,9,26,27,28,29,30] were included in this review of reviews, of which only one was a systematic review [30] and eight were narrative reviews of the literature.

**Figure 1:**
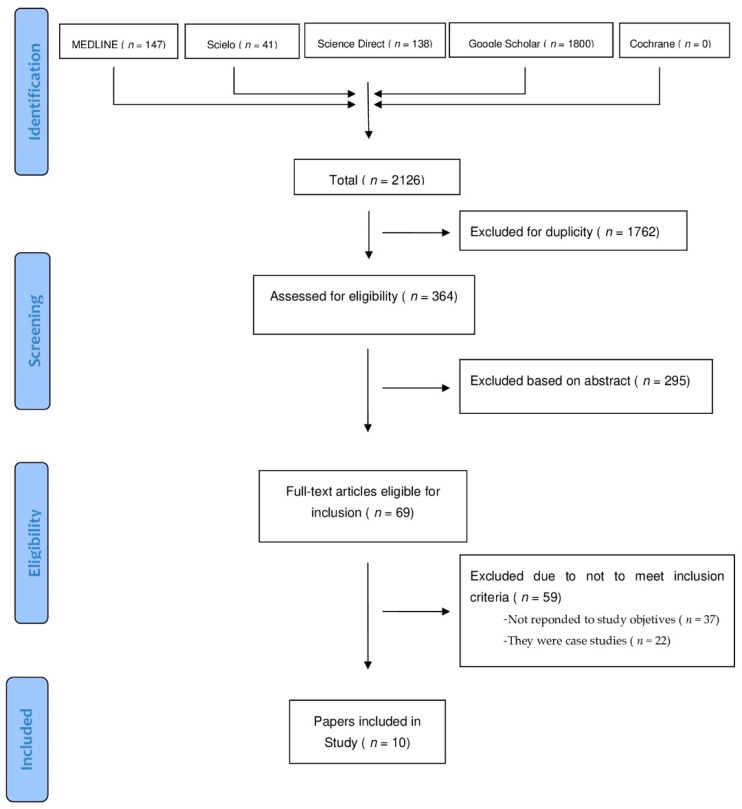
The preferred reporting items for systematic reviews and meta-analyses (PRISMA) flowchart of the conducted search.

Figure 1 shows the PRISMA flowchart and the study selection process.

### 3.2. Methodological Quality

**Figure 2:**
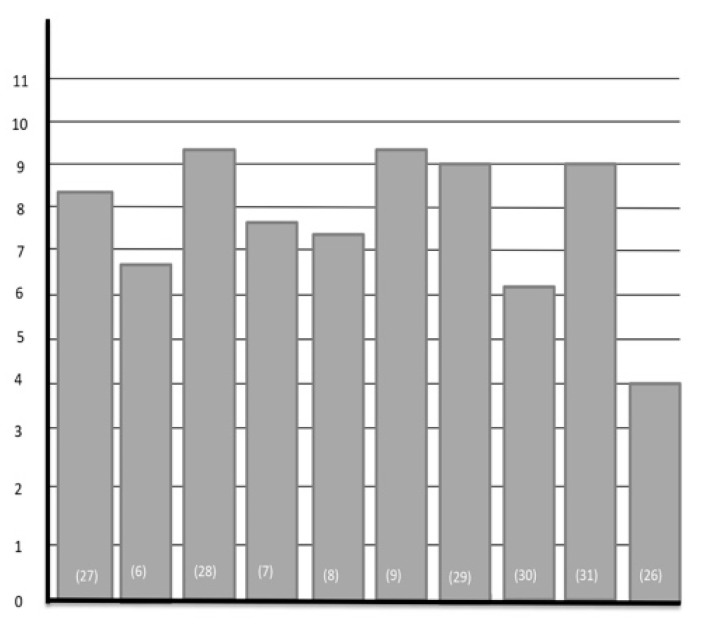
Methodological quality.

Ten reviews were evaluated, 1 systematic review [30] and 9 narrative reviews [6,7,8,9,26,27,28,29], with one being eliminated for low quality, (AMSTAR 1 score < 8) [25]; the rest were of high quality (AMSTAR 1 score ≥ 8). The methodological quality of each review was chosen according to the summary in Figure 2.

### 3.3. Characteristics of the Included Reviews

The reviews were published in 2020, and included one systematic review [30], and eight non-systematic reviews [3,4,5,6,23,24,26,28]. The number of primary studies analyzed in four reviews could not be determined [6,8,9,29]. The number of primary studies that each review included ranged from 7 to 46. The characteristics of the nine reviews there were included are shown in Table 3.

### 3.4. Clinical Manifestations of COVID-19 in the Foot

The most relevant results of the manifestations are shown according to the vascular, integumentary, and neurological systems:

Vascular System:

1. Kawasaki disease, defined as a vasculitis mainly in small and medium arteries, appeared in COVID-19 infections as an erythema, edema, and exanthem in the feet and lower limbs [2,6,8];
2. Acral perniosis lesions or pernio erythema in the acral areas with vesicles or pustules similar to chilblains were the most specific clinical form of all symptoms in the feet and usually appeared in the late stages of the disease and were very common in young patients [1,2,6,10,19];
3. Ischemia and distal necrosis were the most serious complications at the foot level, they are a great risk to patients [10,20].

#### 3.4.1. Integumentary System

The skin manifestations appeared several days after the onset of COVID-19 symptoms, making it difficult to identify their link to COVID-19 [7,17,19]. Vesicular, papulosquamous, papulovesicular, urticarial, and maculopapular (most common) skin breakouts; recurrent herpes; livedo reticularis; and necrosis were the most common skin manifestations [1,6].

#### 3.4.2. Neurological System

When the virus infiltrated to the central nervous system it produced neurological pathologies such as encephalitis, ictus, and polyneuropathy [18]. Neurological manifestations in the lower extremities and foot were related to Guillain-Barré syndrome associated with patients with acute symptoms of COVID-19. Guillain-Barré syndrome can lead to paresis, muscle weakness in the lower extremities (the most common neurological manifestation), and paralysis [11,12]. This syndrome usually occurred 10–21 days after COVID-19 diagnosis. Patients often had paresis, lower limb areflexia, hypoparesis, muscle weakness, ataxia, and difficulty walking [10,11,12]. Table 4 shows the clinical manifestations of COVID-19 in the foot.

## 4. Discussion

The aim of this review of reviews was to analyze reviews about the foot-level manifestations of COVID-19 disease. There were no previous reviews on the specific topic of the foot. This review of reviews identified some reviews that studied clinical manifestations in relation to this disease.

Manifestations in the feet of both children and adults were the inclusion criteria of the reviews. In the child population, one of the most characteristic vascular system manifestations of COVID-19 is Kawasaki disease, a rare, acute, pediatric vasculitis, common at 7 years of age and regardless of sex. Another clinical manifestation is the multisystem inflammatory syndrome, with exacerbation and inflammatory reactions in infants with COVID-19. Recalcati et al. and Benavides et al. both characterize in their studies the appearance of this vasculitis with the presence of edema and exanthem in the lower limbs and feet of children [2,8].

With respect the dermatological lesions on the feet of patients with COVID-19, there were a variety of possibilities. The most common and specific skin manifestations in the feet were pseudo-chilblains or COVID toes, which are related to the vascular problems that COVID-19 can cause, such as vasospasms and inflammation in acral areas, and are unrelated to exposure to cold or moisture. The authors agree on the predominance of onset in young people, of both sexes, and that it is linked to a negative polymerase (known as PCR-). Since the patients with COVID-19 who develop these types of chilblains have mild or asymptomatic clinical courses of infection, which may not generate antibody responses, the chilblains are a subsequent clinical manifestation of COVID-19 [1,6,10,28].

Carrascosa et al. [20] consider that skin manifestations such as chilblains or others, blamed on COVID-19, may be caused by an epidemiological coincidence and not directly attributable to this infection. According to García-Molina et al. [9], the skin signs in the acute phase of COVID-19 are closely related to severe acute cases, highlighting the heterogeneity of skin manifestations both at the general level and at the foot level.

There are some neurological symptoms shown in lower limbs and feet, such as COVID-19-related Guillain-Barré Syndrome; they were found to be suffered by people 60 years of age and older (the pre-pandemic average of this disease being about 40 years of age), with more severe clinical involvement and manifestations in the current cases [7,30]. Guillain-Barré Syndrome is an acute polyradiculopathy characterized by rapidly progressive symmetrical limb weakness, areflexia, sensory symptoms and, in some patients, facial weakness [31]. These neurological symptoms in lower limbs, muscle weakness, and reduced reflexes, all associated with the presence of COVID-19, can lead to and increase the risk of falls in older people suffering from them, with falls in the elderly being considered another complication of neurological origin or even described as a nonspecific symptom of COVID-19 [32]. On the other hand, there are authors who claim that the neuroinvasion capacity of the SARS-CoV-2 virus and its capacity to develop neurological complications [7] is yet to be determined.

This systematic review of reviews has strengths and weaknesses to consider when interpreting results. The main strength of this review of reviews is that it provides a systematic synthesis of the latest scientific evidence on clinical manifestations of COVID-19 at the foot level.

The demand for information is high at a time when evidence is low. As a result, there has been a renaissance in the publication of case studies. This type of research, previously relegated to conventional journals and considered to be a low-level source of evidence, has enabled the quick reporting, publication, and dissemination of much-needed clinical data [33].

For this reason, the decision was made to identify and synthesize recent general evidence based on the review of reviews methodology. An additional problem was the limited existence of systematic reviews, with there being more narrative reviews and no organized pattern in the methodology. Due to the recent onset of this disease and the difficulty of finding studies with acceptable methodological quality and a high-level of evidence design, the research done in this study is inconclusive. Numerous future studies on this subject are needed, as it is essential that health professionals and the general population know the relationship between SARS-CoV-2 infection and the various manifestations in the feet, so that it will be possible to identify such lesions early to establish treatments, mitigate the complications of this infection, and prevent the spread of the virus through asymptomatic infected people.

## 5. Conclusions

In conclusion, foot-level manifestations in relation to COVID-19 are mainly identified by vascular, dermatological, and neurological involvement. More specifically, the manifestations at the vascular level include edemas, exanthems, chilblains, ischemia, and distal necrosis; at the dermatological level they include vesicular skin breakouts, maculopapular, papulosquamous, urticarial, and recurrent herpes; and at the neurological level they include muscle weakness in lower limbs, paresis, areflexias, ataxia, and difficulty walking. The work evaluated does not allow for strong conclusions to be made due to the limited knowledge currently available about the disease and the scarce evidence and methodological quality existing in this regard.

## Figures and Tables

**Table 1:** PEO.

Element	Inclusion Criteria
(P) Population	Adults and children with clinical manifestations on the foot due to COVID-19
(E) Exposure	Exposed to COVID-19
(O) Outcome	Signs and symptoms of COVID-19 in the foot; neurological, vascular, dermatological…

**Table 2:** Search Strings.

Database	Search String	N
PUBMED	(COVID-19 AND FOOT) AND LIMIT TO (HUMANS) AND LIMIT TO (SPANISH OR ENGLISH) AND LIMIT TO (NOT SURGERY) AND TITLE, ABSTRACT AND LIMIT TO (NO STUDY CASES) AND (REVIEW)	147
SCIELO	(COVID-19) AND (CLINICAL MANIFESTATION) AND LIMIT TO (SPANISH OR ENGLISH) AND TITLE, ABSTRACT AND LIMIT TO (NO STUDY CASES) AND (REVIEW)	41
SCIENCE DIRECT	(COVID-19) AND (FOOT MANIFESTATION), AND LIMIT TO (NOT DIABETIC)) AND TITLE, ABSTRACT AND LIMIT TO (NO STUDY CASES) AND (REVIEW)	138
COCHRANE DATABASE SYSTEMATIC REVIEWS	(COVID-19) AND (SIGNS AND SYMPTOMS OR CLINICAL MANIFESTATION) AND (FOOT) AND LIMIT TO (LAST YEAR)	0
GOOGLE ACADEMIC	(COVID-19) AND (CLINICAL MANIFESTATIONS IN LOWER LIMBS) AND LIMIT TO (PUBLICATION YEAR FROM 2020), AND LIMIT TO (NO ANIMALS), AND LIMIT TO (NOT RESPIRATORY, NOT LUNG), AND TITLE, ABSTRACT AND LIMIT TO (NO STUDY CASES) AND (REVIEW)	1800

**Table 3:** Characteristics of the included reviews.

Review (Year)	Objective	Type of Review	Number of Final Studies Included	Year of Studies Included	Provides Search Terms	Language Restrictions	Quality Assessment	Synthesis of Evidence
Jia et al. (2020) [26]	Describe cutaneous symptoms associated with COVID-19 presentation	Preliminary review	46	2020	YES	YES	YES	Narrative and tabular
Santos et al. (2020) [27]	Analyze the current scientific literature to document in an integrative review the main findings that correlate Kawasaki disease (KD) to COVID-19	Integrative literature review	7	2020	YES	NO	YES	Narrative
Morel-Ayala et al. (2020) [6]	Review of SARS-Cov-2 extrapulmonary manifestations	Literature review	Not specified	2020	YES	Not specified	Not specified	Narrative
Zayas-Fundora et al. (2020) [7]	Describe neurological complications caused by SARS-CoV-2	Bibliographic review	31	2020	YES	YES	YES	Narrative
Benavides-Reina et al. (2020) [8]	Differences between children and adults with COVID-19, clinical manifestations (emphasizing multi-system post-inflammatory syndrome), diagnostic tests, and the role of children in community transmission of infection	Review	Not specified	2020	YES	Not specified	Not specified	Narrative
García-Molina et al. (2020) [9]	Provide an overview of skin manifestations in patients with COVID-19	Bibliographic review	Not specified	2020	YES	YES	Not specified	Narrative and tabular
Gottlieb et al. (2020) [28]	Summarize dermatologic manifestations and complications associated with COVID-19 with an emphasis on emergency medicine clinicians	Literature review	41	2020	YES	Not specified	Not specified	Narrative
Yamamoto et al. (2020) [29]	Summarize the main aspects underlying the new coronavirus SARS-CoV-2	Narrative review	Not specified	2020	YES	Not specified	Not specified	Narrative
Trujillo-Gittermann et al. (2020) [31]	Analyze the available evidence that associates Guillain-Barré syndrome with COVID-19	Systematic review	24	2020	YES	Not specified	YES	Narrative and tabular

**Table 4:** Clinical manifestations of COVID-19 in the foot.

Lesion	Population	Appearance	Evolution	Prognosis	Prevalence
Foot edema associated with Kawasaki Disease [2,8]	Infancy	Early, 30 days before diagnosis of KD	-	-	60.7%
Exanthem in the feet associated with Kawasaki Disease [2,8]	Infancy	Early, 30 days before diagnosis of KD	-	-	55.6%
Skin rash associated with multisystem inflammatory syndrome [8]	Children and teenagers	After >3 days with fever	-	-	56.3%
Chilblains or perniosis [1,6,10]	Young people	Late stage of the disease	Usually lasted 14 days	Good	19%
Ischemia and distal necrosis [10]	Adults	-	-	Bad	6%
Vesicular breakouts [1,6,10]	Adults, small percentage in children	Prior to other symptoms or early stages of the disease	10 day duration	Bad	9%
Urticarial lesions [1,6,10]	Children	Within 3 days of diagnosis and along with respiratory symptoms	Disappeared after 8 days	Bad	19%
Maculopapular lesions [1]	-	Along with respiratory symptoms	Lasted 10–14 days	-	22.7%
Papulosquamous breakouts [9]	Adults	-	-	-	47%
Recurrent herpes [9]	Adults	-	-	-	-
Muscle weakness in lower limbs, paresis, areflexias, ataxia, and difficulty walking through COVID-associated Guillain-Barré Syndrome [7,10,11,12]	Adults (more common in 35–50 years of age)	10–21 days after COVID-19 diagnosis	-	Bad	-

## Data Availability

Not applicable.
